# Oral anticoagulants increased 30-day survival in sepsis patients complicated with atrial fibrillation: a retrospective analysis from MIMIC-IV database

**DOI:** 10.3389/fcvm.2024.1322045

**Published:** 2024-01-18

**Authors:** Gaoyuan Ge, Dan Bo, Rongli Jiang, Wei Zhao, Yao Lu

**Affiliations:** ^1^ Department of Cardiology, The Affiliated Hospital of Yangzhou University, Yangzhou, Jiangsu, China; ^2^ Department of Cardiology, The First Affiliated Hospital of Nanjing Medical University, Nanjing, Jiangsu, China; ^3^ Department of Geriatric, The Affiliated Hospital of Yangzhou University, Yangzhou, Jiangsu, China; ^4^ Department of Cardiology, Xuzhou Central Hospital, Xuzhou Institute of Cardiovascular Disease, Xuzhou Clinical School of Nanjing Medical University, Xuzhou, Jiangsu, China

**Keywords:** atrial fibrillation, sepsis, warfarin, novel oral anticoagulant, in-hospital mortality

## Abstract

**Background:**

The severity of sepsis is associated with systemic clotting activation. Atrial fibrillation (AF) is the most commonly observed arrhythmia in patients with sepsis and can lead to a poor prognosis. The aim of this study is to elucidate the association between oral anticoagulants and survival from septic patients complicated with AF.

**Methods:**

The data of 8,828 septic patients, including 2,955 AF and 5,873 without AF, were all originated from the Medical Information Mart for Intensive Care IV (MIMIC-IV) database. Patients with sepsis and AF are divided into OAC- group (*n* = 1,774) and OAC+ group (*n* = 1,181) based on OAC therapy. Septic patients with no AF were considered as the control group (*n* = 5,873, sepsis and no AF group). The main outcome endpoint was the survival rate of 30 day. The secondary outcome endpoint was the length of stay (LOS) from intensive care unit and hospital. Propensity score matching (PSM) was used to adjust the influence of superfluous factors, and a restricted mean survival time (RMST) analysis was used for calculating the benefit of survival time and survival rate. Analysis including univariate and multivariate logistic regression analysis was conducted to find prognosis-related predictors.

**Results:**

After PSM, the OAC+group had a higher 30-day survival rate compared to the OAC- group (81.59% vs. 58.10%; *P* < 0.001) in the ICU. Despite the higher survival, the hospital LOS (14.65 days vs. 16.66 days; *P* = 0.15) and ICU LOS (6.93 days vs. 5.92 days; *P* = 0.02) were prolonged at OAC+ group than OAC- group. No difference was found in survival rate of 30 day between the sepsis patients using warfarin and patients using NOAC (85.60% vs. 79.84%, *P* = 0.12). The sepsis patients using warfarin had a prolonged LOS in ICU and hospital compared with the sepsis patients using NOAC. In the vasopressor subgroup, patients who received NOAC therapy were associated with a reduced 30-day survival rate (73.57% vs. 84.03%; *P* = 0.04) and reduced LOS in ICU and hospital than those on warfarin therapy.

**Conclusion:**

This study demonstrated that oral anticoagulants may increase the 30-day survival rate of patients with sepsis and AF.

## Introduction

Atrial fibrillation (AF) is the most commonly observed arrhythmia from people who suffer from sepsis (1). There was strong association between sepsis and AF (2). Sepsis is characterized by systemic inflammation activation, intravascular volume overload, excessive adrenergic stimulation, and impairment of cardiovascular system. all factors above can contribute to abnormal development of atrial electrophysiology, resulting in the occurence of AF (3–5).

Sepsis is responsible for the majority of mortality in the ICU (6). Patients who have experienced severe shock and atrial fibrillation tend to cause the aggravation of illness and have poor prognosis (7). Sepsis patients with new-onset AF had higher risk of death than sepsis patients with no AF (8).

The severity of sepsis is highly associated with systemic clotting activation. Enough evidence was found between hemostasis and inflammation and hemostasis is important for the development of illness in patients with sepsis (9). Sepsis patients and DIC may develop severe complications, which can result in failure of multiple organs (10, 11). The evidence was cumulating for beneficial effect of anticoagulation therapy in sepsis patients (12). In addition, more reseach demonstrate that sepsis patients need to prevent venous thrombosis (13).

Novel oral anticoagulants (NOAC) and warfarin are the most common drugs used in clinical anticoagulation therapy, but their effects on the prognosis of sepsis complicated by atrial fibrillation have rarely been studied. This study aimed to better interpret the deep relationship between anticoagulation therapy (NOAC and warfarin) and its impact on prognosis of sepsis and AF patients.

## Methods

### Study population

**Figure 1:**
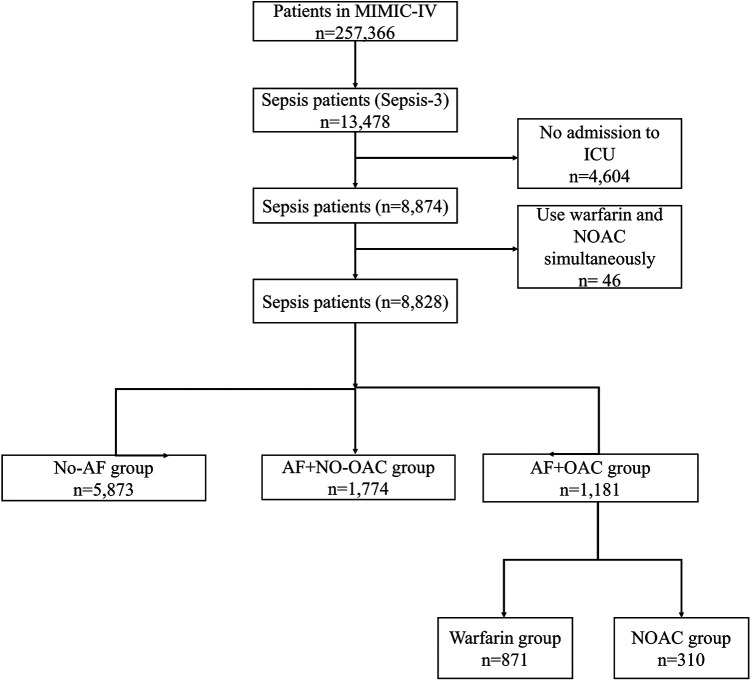
Flow diagram of the selection of eligible patients.

This study was a retrospective observational study. The data originated from the MIMIC-IV database (14, 15). This database was identified by the Massachusetts Institute of Technology. The single-center database had 257,366 individuals, including 13,478 sepsis patients according to the International Classification of Diseases (ICD)-9/10 code. We collect patients who were in ICU. The criteria of exclusion as folows: 1) Patients who were not in ICU; 2) Patients who took warfarin in combination with new oral anticoagulants during hospitalization. Of 13,478 patients with sepsis, 4,604 patients who were not in ICU were excluded. Remaining 8,828 patients were included to next analysis (Figure 1). 2,995 (33.9%) septic patients were complicated with AF. Of them, 1,181 (39.4%) patients were treated with OAC (NOAC or warfarin) and included in the OAC+ group. Remaining 1,774 septic patients complicated with AF who had not received OAC during hospitalization included in OAC- group. 5,873 patients with sepsis but no AF included in the control group (Sepsis, no AF).

Patients who had received vasopressor therapy in the hospital were included in a vasopressor subgroup indicating the severe condition of sepsis. The vasopressor subgroup consisted of septic patients who received the vasopressors therapy (norepinephrine, phenylephrine, epinephrine, dopamine, or dobutamine) during hospitalization.

### Main and secondary outcomes

The main outcome endpoint was the survival rate of 30 day. The secondary outcome endpoint was the length of stay (LOS) from intensive care unit and hospital.

### Establishment of Kaplan–Meier (KM) survival curve

The definition of Kaplan–Meier (KM) survival curve is included in this study (16). We used the R package (“survminer” (https://rpkgs.datanovia.com/survminer/index.html) and “survival” (17)) to establish curves of survical from two groups (OAC+ and the OAC- groups; warfarin and NOAC groups) and compared them in pairs.

### Propensity score matching (PSM)

PSM was utilized to eliminate confounding factors from the OAC+ and the OAC- groups, as well as warfarin and NOAC groups. After PSM, standardized mean differences (SMD) were utilized to prove that confounding factors were balanced between the two groups (18).

### Statistical analysis

Baseline characteristics of the study patients are showed as mean ± SD or percentages for continuous and categorical variables. *T*-test was used to compare the patients' characteristics between two groups. The pairwise comparison between the three groups was performed using ANOVA. Variables that is significant in univariate analysis were included in multivariate analysis.

## Results

### Baseline characteristics for all patients

Demographic characteristics for all sepsis patients are detailed in Supplementary Table S1 and the *P*-values for pairwise comparison are shown in Supplementary Table S2. The mean age of the study patients was 67.50 ± 16.20 years, 4,006 (45.38%) were female, and 863 (9.78%) had continuous renal replacement therapy (CRRT). 30-day mortality was significantly higher for OAC- group (47.75%) than that of OAC+ group (17.44%) and sepsis, no AF group (26.82%) (*P* all <0.001). The occurrence of shock induced by sepsis was also significantly higher for OAC- group (31.74%) compared with that of OAC+ group (22.18%) as well as sepsis, no AF group (27.15%) (*P* all <0.001). The 30-day survival curve suggested significant differences among the three groups (Supplementary Figure S1) (*P* < 0.001). OAC- group had higher ICU LOS and hospital than OAC+ as well as sepsis, no AF group (control group) (*P* all <0.001).

### 30-day survival and LOS in ICU & hospital after PSM

**Figure 2:**
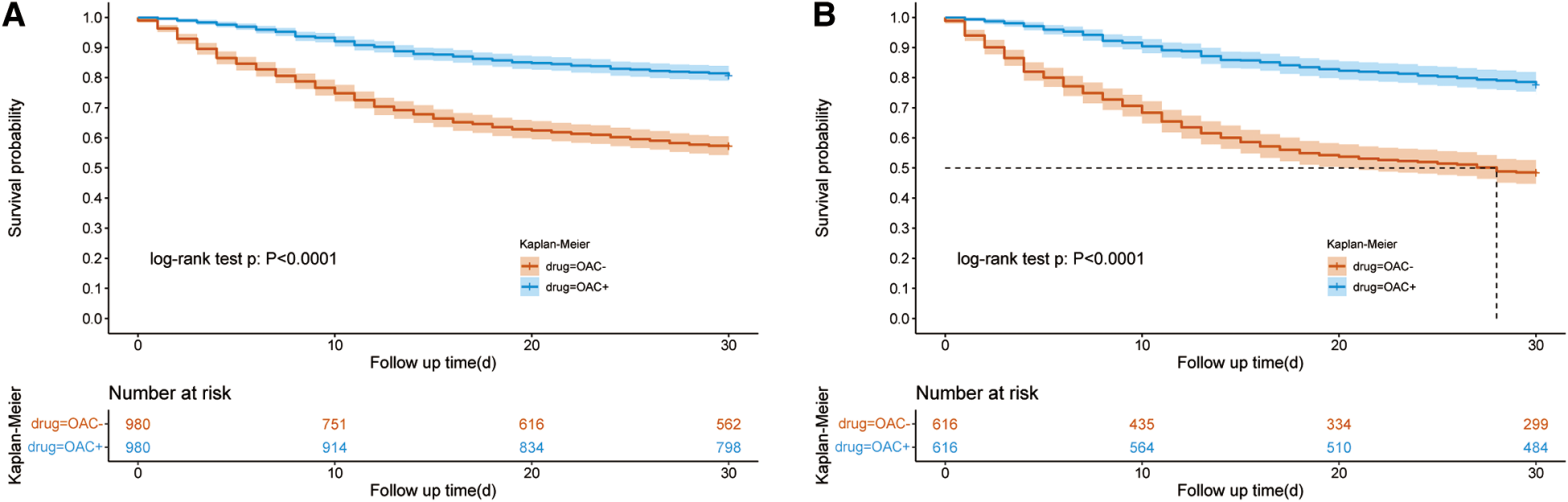
Kaplan–Meier survival curve of the OAC + and OAC- groups. Figure 2A showed the Kaplan–Meier survival curve of the OAC+ and OAC- groups after PSM in all patients; Figure 2B showed the Kaplan–Meier survival curve of the OAC+ and OAC- groups after PSM in the vasopressor subgroup. OAC, oral anticoagulants; AF, atrial fibrillation; PSM, propensity score matching.

After PSM (1:1), the SMD of variables are totally smaller than 0.1, showing that The confounding factors of the two groups were basically balanced (Table 1). We performed a multifactor analysis of post-PSM data to identify factors that potentially influence 30-day mortality (Supplementary Figure S2A). The KM curve showed that the OAC therapy had an rising survival rate of 30 day (81.59% vs. 58.10%; *P* < 0.001) (Figure 2A). Prolonged ICU LOS (Length of Stay) (6.93 days vs. 5.92 days; *P* = 0.02) and shortened hospital LOS (14.65 days vs. 16.66 days; *P* = 0.15) were found in the OAC+ group compared to the OAC- group (Table 2A), the difference in ICU LOS was significant and the difference in hospital LOS was non-significant.

**Table 1:** Baseline table of demographic characteristics after PSM.

	Sepsis and AF, OAC-	Sepsis and AF, OAC+	SMD
	*n* = 980	*n* = 980	
Age (Yr)	76.00 ± 11.59	76.16 ± 10.95	0.015
BMI (kg/m^2^)	29.37 ± 8.66)	28.99 ± 7.66	0.047
Female (%)	381 ± 40.3)	388 ± 41.1	0.015
Hemoglobin (g/dl)	10.52 ± 2.24	10.55 ± 2.24	0.015
Platelets (×10^9^/L)	225.12 ± 127.46	222.72 ± 124.87	0.019
Neutrophils (×10^9^/L)	81.45 ± 11.39	80.65 ± 11.45	0.069
First time heart rate (bpm)	95.46 ± 21.61	95.66 ± 22.89	0.009
First time SBP (mmHg)	115.70 ± 24.14	115.45 ± 24.08	0.01
First time temperature (°C)	36.85 ± 0.85	36.84 ± 0.82	0.009
First time spo2 (%)	95.92 ± 4.40	95.82 ± 4.24	0.024
ALP (IU/L)	137.22 ± 129.36	135.56 ± 95.95	0.015
AST (IU/L)	197.80 ± 666.97	188.42 ± 690.32	0.014
Total bilirubin (mg/dl)	1.61 ± 2.10)	1.51 ± 1.85	0.05
Creatinine (mg/dl)	1.92 ± 1.64	1.99 ± 1.70	0.041
INR	2.03 ± 1.72	2.17 ± 1.41	0.083
PT (s)	21.93 ± 18.08	23.29 ± 14.94	0.082
PTT (s)	40.07 ± 23.57	40.89 ± 23.39	0.035
SOFA score	7.35 ± 3.79	7.57 ± 4.01	0.057
GCS score	11.72 ± 3.81	11.55 ± 3.77	0.046
CRRT (%)	99 (10.5)	113 (12.0)	0.047
Beta blocker (%)	744 (78.7)	729 (77.1)	0.038
CKD (%)	356 (37.7)	378 (40.0)	0.048
COPD (%)	101 (10.7)	101 (10.7)	<0.001
CVD (%)	29 (3.1)	30 (3.2)	0.006
DM (%)	336 (35.6)	322 (34.1)	0.031
HP (%)	190 (20.1)	178 (18.8)	0.032
Gastrointestinal bleeding (%)	26 (2.8)	28 (3.0)	0.013
Cerebral hemorrhage (%)	3 (0.3)	4 (0.4)	0.017
Use of heparin (%)	536 (56.7)	556 (58.8)	0.043

Data are presented as mean ± SD, median (25th–75th percentile) or median (percentile). NO-OAC, no use of oral anticoagulants; OAC, oral anticoagulants; INR, international normalized ratio; PT, prothrombin time; PTT, partial thromboplastin time; SOFA, sequential organ failure assessment; GCS, glasgow coma scale; SBP, systolic blood pressure; NOAC, novel oral anticoagulants; CRRT, continuous renal replacement therapy; CKD, chronic kidney disease; COPD, chronic obstructive pulmonary disease; CVD, cardiovascular disease; DM, diabetes mellitus; HP, hypertension.

**Table 2A:** Outcomes for all patients.

	Sepsis and AF, OAC- (*n* = 1,774)		Sepsis and AF, OAC + (*n* = 1,181)		Difference (95% CI)	*P*
	Values	95% CI	Values	95% CI		
After PSM (1:1)						
30-day survival rate (%)	58.10		81.59			<0.001
LOS ICU (days)	5.92	5.29–6.55	6.93	6.36–7.50	1.01 (0.16–1.83)	0.02
LOS hospital (days)	14.65	12.07–17.23	16.66	15.72–17.60	2.01 (−0.74–4.75)	0.15

**Table 2B:** Outcomes for vasopressor subgroup.

	Sepsis and AF, OAC- (*n* = 1,166)		Sepsis and AF, OAC + (*n* = 713)		Difference (95%CI)	*P*
	Values	95% CI	Values	95% CI		
After PSM (1:1)						
30-day survival rate (%)	49.57		78.47			<0.001
LOS ICU (days)	7.23	6.60–7.86	9.39	8.57–10.21	2.16 (1.12–3.20)	<0.001
LOS hospital (days)	13.92	12.84–14.99	18.90	17.58–20.21	4.98 (3.28–6.68)	<0.001

LOS ICU: Length of ICU stay; LOS Hospital: Length of hospital stay; PSM: propensity score matching.

### 30-day survival and LOS in vasopressor subgroup after PSM

The use of vasopressor drugs in sepsis is a sign of severe disease, and we analyzed this subset of patients separately. We performed a multifactor analysis of post-PSM data to identify factors that potentially influence 30-day mortality in vasopressor group (Supplementary Figure S2B). After PSM, the KM curve showed that the OAC therapy was associated with an increased 30-day survival rate (78.47% vs. 49.57%; *P* < 0.001) in the vasopressor subgroup (Figure 2B). The LOS in ICU was reduced in the OAC+ group than in the OAC- group (9.39 days vs.7.23 days; *P* < 0.001) (Table 2B).

### 30-day survival and LOS between warfarin group and NOAC group

**Figure 3:**
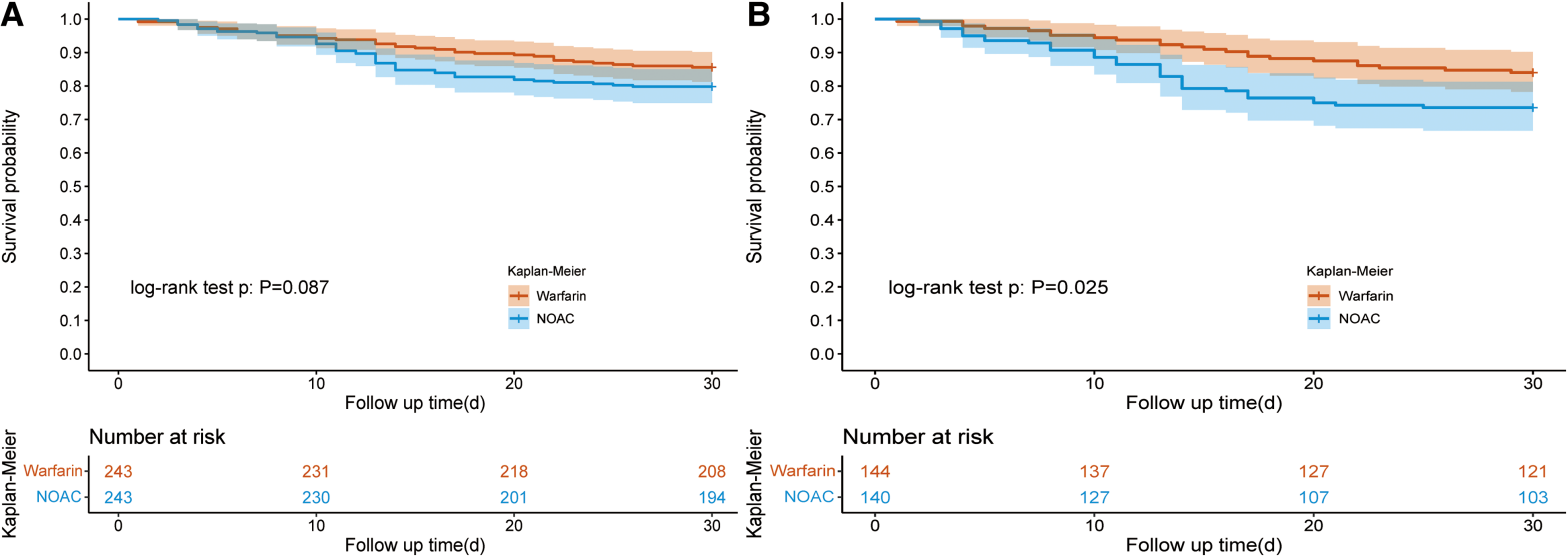
Kaplan–Meier survival curve of the warfarin and NOAC groups. Figure 3A showed the Kaplan–Meier survival curve of the warfarin and NOAC groups after PSM in all patients; Figure 3B showed the Kaplan–Meier survival curve of the warfarin and NOAC groups after PSM in the vasopressor subgroup. NOAC, novel oral anticoagulants; PSM, propensity score matching.

For oral anticoagulants, it mainly includes warfarin and NOAC. Of 1,181 patients taking anticoagulants, 871 used warfarin, and 310 used NOAC. After PSM (1:1) (Supplementary Table S2), the KM curve showed no difference was found in the 30-day survival rate from the warfarin and NOAC group (85.60% vs. 79.84%, *P* = 0.12) (Table 3A and Figure 3A). The hospital LOS (18.13 days vs. 13.92 days; *P* = 0.002) and ICU LOS (6.75 days vs. 4.84 days; *P* = 0.004) were prolonged in the warfarin group than the NOAC group.

**Table 3A:** Outcomes for all patients.

	Warfarin group (*n* = 871)		NOAC group (*n* = 310)		Difference (95% CI)	*P*
	Values	95% CI	Values	95% CI		
After PSM (1:1)						
30-day survival rate (%)	85.60%		79.84%			0.12
LOS ICU (days)	6.75	5.67–7.84	4.84	4.11–5.56	−1.92 (−3.22–0.61)	0.004
LOS hospital (days)	18.13	15.76–20.49	13.92	12.65–15.19	−4.21 (−6.89–1.53)	0.002

**Table 3B:** Outcomes for vasopressor subgroup.

	Warfarin group (*n* = 538)		NOAC group (*n* = 175)		Difference (95% CI)	*P*
	Values	95% CI	Values	95% CI		
After PSM (1:1)						
30-day survival rate (%)	84.03		73.57			0.04
LOS ICU (days)	9.43	7.78–11.08	6.46	5.41–7.50	−2.97 (−4.93–1.01)	0.003
LOS hospital (days)	20.66	17.26–24.06	14.82	13.09–16.56	−5.84 (−9.67–2.01)	0.003

LOS ICU, length of ICU stay; LOS Hospital, length of hospital stay; PSM, propensity score matching.

In the vasopressor subgroup, patients who received NOAC therapy were associated with a reduced 30-day survival rate (73.57% vs. 84.03%; *P* = 0.04) than the warfarin group after PSM (Table 3B). The ICU LOS (9.43 days vs. 6.46 days, *P* = 0.003) and hospital LOS (20.66 days vs. 14.82 days; *P* = 0.003) both were prolonged in the warfarin group compared to the NOAC group (Table 3B).

### Risk factors related to all-cause mortality in sepsis combined with AF

**Figure 4:**
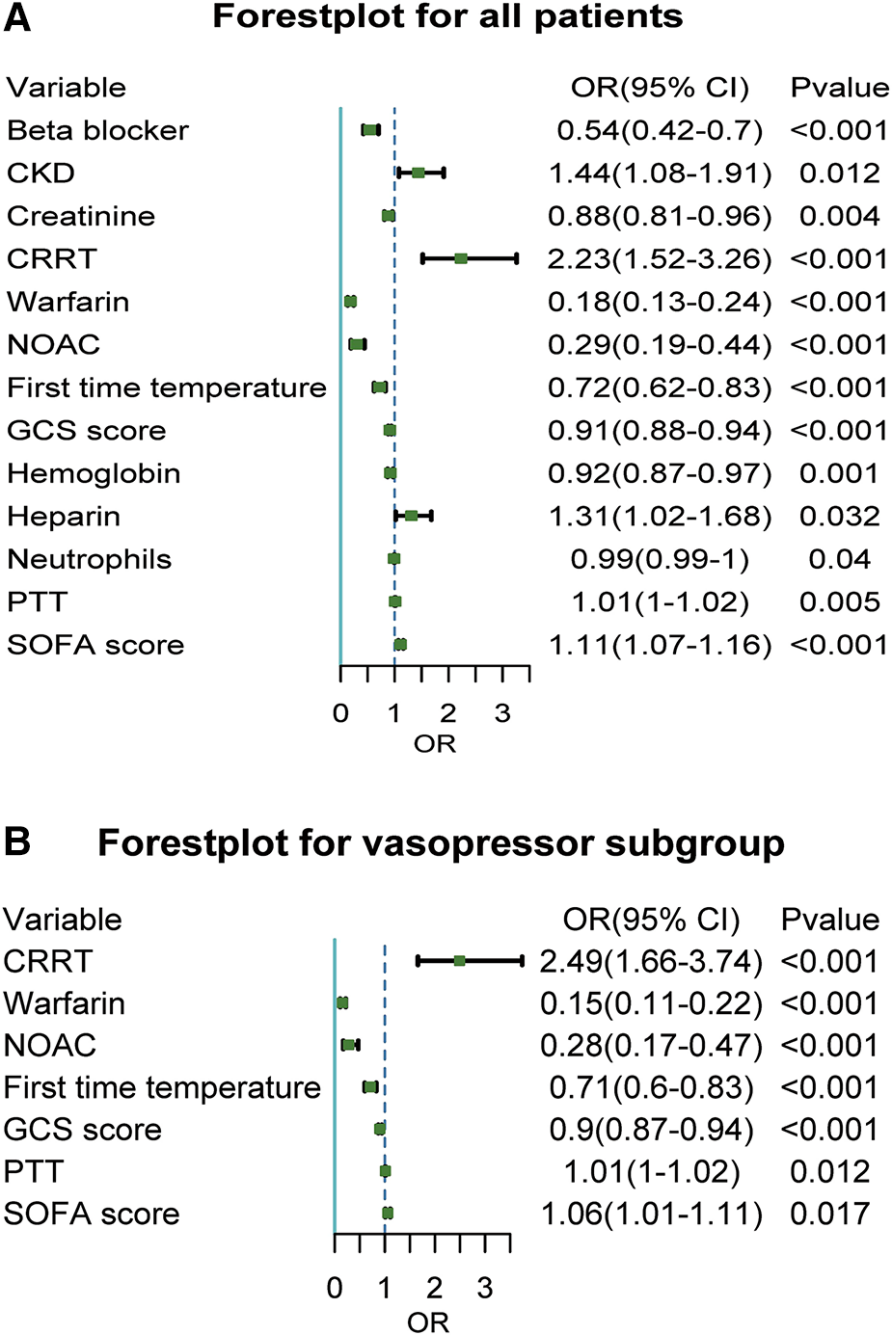
Forest plot of related factors affecting death for sepsis with atrial fibrillation patients. Figure 4A showed a Forest plot of related factors affecting death for sepsis with atrial fibrillation for all patients; Figure 4B showed a Forest plot of related factors affecting death for sepsis with atrial fibrillation for the vasopressor subgroup. CKD, chronic kidney disease; CRRT, continuous renal replacement therapy; NOAC, novel oral anticoagulants; GCS, glasgow coma scale; SOFA, sequential organ failure assessment.

We investigated the risk factors related to mortality in sepsis and AF patients. Chronic Kidney disease (CKD) significantly increased all-cause mortality of sepsis patients with an adjusted OR of 1.44 (95% CI, 1.08–1.91; *P* = 0.012) (Figure 4A). CRRT during hospitalization significantly increased all-cause mortality with an adjusted OR of 2.23 (95% CI, 1.52–3.26; *P* < 0.001) and 2.49 (95% CI, 1.66–3.74; *P* < 0.001) in vasopressor subgroup. Use of warfarin and NOAC both significantly reduced all-cause mortality with an adjusted OR of 0.18 (95% CI, 0.13–0.24; *P* < 0.001) and 0.29 (95% CI, 0.19–0.44; *P* < 0.001) and verified in vasopressor subgroup. The higher temperature on the first day of hospitalization and lower GCS score reduce all-cause mortality in all patients, including the vasopressor subgroup. In contrast, higher SOFA scores increase all-cause mortality in all patients, including the vasopressor subgroup.

## Discussion

In this study, we revealed that oral anticoagulants can increase the 30-day survival rate but prolonged LOS in ICU in sepsis patients complicated with AF after matching heparin use. No difference was found in survival rate of 30 day between warfarin and NOAC groups. However, warfarin showed significantly increased LOS in hospitals and ICU than NOAC in the vasopressor subgroup. CRRT and CKD were independent risk factors for all-cause mortality.

Among patients with severe sepsis, patients with new-onset AF were at increased risk of in-hospital stroke and death compared with patients with no AF and patients with preexisting AF (19, 20). The question of whether patients with sepsis and atrial fibrillation should receive anticoagulation therapy is still controversial (21).We observed that anticoagulant therapy effectively reduces 30-day mortality in septic and AF patients. Allan J Walkey et al. proved that after hospitalization with new-onset AF during sepsis, oral anticoagulation use was uncommon and associated with potentially higher stroke/TIA risk (22). Umemura et al. reported a meta-analysis showing that anticoagulant therapy could improve the mortality in sepsis-induced DIC patients. However there was no beneficial effect on survival in sepsis patients (23). The foundation for anticoagulant method comes from increasing evidence suggesting beneficial effects in sepsis patients (24). More and more studies had reported the important relationship between anticoagulant therapy and inflammation in sepsis patients (24). Damage-associated molecular patterns (DAMPs), has been proved to participate in the pathogenesis of sepsis.

We have not found any difference in survival rate of 30 day between warfarin and NOAC groups in sepsis and AF patients. However, some studies showed that NOAC reduces mortality in AF patients compared with warfarin (25, 26). This may be due to different subjects. In our study, the patients had severe sepsis and AF, while the previous studies included AF patients without sepsis.

Similarly, prolonged LOS in hospital and ICU were found in the OAC+ group compared to the OAC- group, which was different from the previous study (23). In fact, heparin, not NOAC or warfarin, was used for reducing LOS in hospitals of sepsis patients. Studies have shown that NOAC has a shorter hospital stay than warfarin in AF patients with higher body weight or post-cardiac surgery (27, 28). This may contribute to lower complications (such as bleeding) in NOAC compared with warfarin.

A similar conclusion was gained that NOAC had no difference in survival rate compared with warfarin but reduced LOS in hospitals in post-cardiac surgery atrial fibrillation patients (29). We have found that warfarin therapy prolonged LOS in ICU and hospitals compared to NOAC, which was similar with previous studies.

We also found that CRRT and CKD were independent risk factors for all-cause mortality in sepsis patients with AF, which was consistent with previous studies (30).

This study has several limitations. Firstly, PSM analysis may reduce the size of sample. The distribution of matched datasets was less than the main dataset; Secondly, although the PSM were to reduce the confounders, some confounders may not be measured in this study. Thirdly, the data about the outcome of bleeding and embolism after anticoagulant therapy is missing in the MIMIC public database. Fourth, as a retrospective observational study, the credibility of this study needs to be further confirmed in prospective studies. Fively, due to the use of a shared database for analysis, there are too many messy diagnostic codes related to DIC, so it is impossible to assess that the patients who were on vasopressor therapy is DIC. Sixly, the basis that patients with atrial fibrillation and sepsis not treated with an anticoagulant is unclear. Finally, the work completely lacks a reference to which patients had newly diagnosed atrial fibrillation and which ones were previously diagnosed with atrial fibrillation.

## Conclusion

This retrospective study confirms that oral anticoagulants can increase the 30-day survival rate in sepsis patients complicated with AF. No significant difference was found in the 30-day survival rate between warfarin and NOAC groups. Warfarin was associated with prolonged LOS in ICU and hospital compared with NOAC.

## Data Availability

The original presented in the study are included in the article/Supplementary Materials, further inquiries can be directed to the corresponding authors.
