# Supplementary material for: Oral anticoagulants increased 30-day survival in sepsis patients complicated with atrial fibrillation: a retrospective analysis from MIMIC-IV database
Source: Front Cardiovasc Med. 2024 Jan 18;11:1322045. doi: 10.3389/fcvm.2024.1322045 (PMC10830619; doi:10.3389/fcvm.2024.1322045)
Supplement: Supplementary file 1 [file Table1.docx]

**Table 1: Baseline table of demographic characteristics after PSM**

|  | **Sepsis and AF, OAC-** | **Sepsis and AF, OAC+** | **SMD** |
| --- | --- | --- | --- |
|  | **n=980** | **n=980** |  |
| Age (Yr) | 76.00±11.59 | 76.16±10.95 | 0.015 |
| BMI (kg/m^2) | 29.37±8.66) | 28.99±7.66 | 0.047 |
| Female (%) | 381±40.3) | 388±41.1 | 0.015 |
| Hemoglobin (g/dL) | 10.52±2.24 | 10.55±2.24 | 0.015 |
| Platelets (*10^9/L) | 225.12±127.46 | 222.72±124.87 | 0.019 |
| Neutrophils (*10^9/L) | 81.45±11.39 | 80.65±11.45 | 0.069 |
| First time heart rate (bpm) | 95.46±21.61 | 95.66±22.89 | 0.009 |
| First time SBP (mmHg) | 115.70±24.14 | 115.45±24.08 | 0.01 |
| First time temperature (℃) | 36.85±0.85 | 36.84±0.82 | 0.009 |
| First time spo2 (%) | 95.92±4.40 | 95.82±4.24 | 0.024 |
| ALP (IU/L) | 137.22±129.36 | 135.56±95.95 | 0.015 |
| AST (IU/L) | 197.80±666.97 | 188.42±690.32 | 0.014 |
| Total bilirubin (mg/dl) | 1.61±2.10) | 1.51±1.85 | 0.05 |
| Creatinine (mg/dl) | 1.92±1.64 | 1.99±1.70 | 0.041 |
| INR | 2.03±1.72 | 2.17±1.41 | 0.083 |
| PT (s) | 21.93±18.08 | 23.29±14.94 | 0.082 |
| PTT (s) | 40.07±23.57 | 40.89±23.39 | 0.035 |
| SOFA score | 7.35±3.79 | 7.57±4.01 | 0.057 |
| GCS score | 11.72±3.81 | 11.55±3.77 | 0.046 |
| CRRT (%) | 99 (10.5) | 113 (12.0) | 0.047 |
| Beta blocker (%) | 744 (78.7) | 729 (77.1) | 0.038 |
| CKD (%) | 356 (37.7) | 378 (40.0) | 0.048 |
| COPD (%) | 101 (10.7) | 101 (10.7) | <0.001 |
| CVD (%) | 29 (3.1) | 30 (3.2) | 0.006 |
| DM (%) | 336 (35.6) | 322 (34.1) | 0.031 |
| HP (%) | 190 (20.1) | 178 (18.8) | 0.032 |
| Gastrointestinal bleeding (%) | 26 (2.8) | 28 (3.0) | 0.013 |
| Cerebral hemorrhage (%) | 3 (0.3) | 4 (0.4) | 0.017 |
| Use of heparin (%) | 536 (56.7) | 556 (58.8) | 0.043 |

Data are presented as mean ± SD, median (25th–75th percentile) or median (percentile). Abbreviations：NO-OAC：no use of oral anticoagulants; OAC: oral anticoagulants; INR: International Normalized Ratio; PT: prothrombin time; PTT: partial thromboplastin time; SOFA: sequential organ failure assessment; GCS: glasgow coma scale; SBP: systolic blood pressure; NOAC: novel oral anticoagulants; CRRT: continuous renal replacement therapy; CKD: chronic kidney disease; COPD: chronic obstructive pulmonary disease; CVD: cardiovascular disease; DM: diabetes mellitus. HP: hypertension.
