# Supplementary material for: Oral anticoagulants increased 30-day survival in sepsis patients complicated with atrial fibrillation: a retrospective analysis from MIMIC-IV database
Source: Front Cardiovasc Med. 2024 Jan 18;11:1322045. doi: 10.3389/fcvm.2024.1322045 (PMC10830619; doi:10.3389/fcvm.2024.1322045)
Supplement: Supplementary file 2 [file Table2.docx]

|  | Sepsis and AF, OAC- (n=1,774) | | Sepsis and AF, OAC+ (n=1,181) | | Difference  （95%CI） | P |
| --- | --- | --- | --- | --- | --- | --- |
|  | Values | 95%CI | Values | 95%CI |  |  |
| After PSM (1:1) |  | | | | | |
| 30-day survival rate (%) | 58.10 |  | 81.59 |  |  | <0.001 |
| LOS ICU (days) | 5.92 | 5.29-6.55 | 6.93 | 6.36-7.50 | 1.01 (0.16-1.83) | 0.02 |
| LOS Hospital (days) | 14.65 | 12.07-17.23 | 16.66 | 15.72-17.60 | 2.01 (-0.74-4.75) | 0.15 |

**Table2a: Outcomes for all patients**

**Table2b: Outcomes for vasopressor subgroup**

|  | Sepsis and AF, OAC- (n=1166) | | Sepsis and AF, OAC+ (n=713) | | Difference  （95%CI） | P |
| --- | --- | --- | --- | --- | --- | --- |
|  | Values | 95%CI | Values | 95%CI |  |  |
| After PSM (1:1) |  | | | | | |
| 30-day survival rate (%) | 49.57 |  | 78.47 |  |  | <0.001 |
| LOS ICU (days) | 7.23 | 6.60-7.86 | 9.39 | 8.57-10.21 | 2.16 (1.12-3.20) | <0.001 |
| LOS Hospital (days) | 13.92 | 12.84-14.99 | 18.90 | 17.58-20.21 | 4.98 (3.28-6.68) | <0.001 |

LOS ICU: Length of ICU stay; LOS Hospital: Length of hospital stay; PSM: propensity score matching.
