# Supplementary material for: Oral anticoagulants increased 30-day survival in sepsis patients complicated with atrial fibrillation: a retrospective analysis from MIMIC-IV database
Source: Front Cardiovasc Med. 2024 Jan 18;11:1322045. doi: 10.3389/fcvm.2024.1322045 (PMC10830619; doi:10.3389/fcvm.2024.1322045)
Supplement: Supplementary file 3 [file Table3.docx]

|  | Warfarin group (n=871) | | NOAC group (n=310) | | Difference  （95%CI） | P |
| --- | --- | --- | --- | --- | --- | --- |
|  | Values | 95%CI | Values | 95%CI |  |  |
| After PSM (1:1) |  | | | | | |
| 30-day survival rate (%) | 85.60% |  | 79.84% |  |  | 0.12 |
| LOS ICU (days) | 6.75 | 5.67-7.84 | 4.84 | 4.11-5.56 | -1.92 (-3.22--0.61) | 0.004 |
| LOS Hospital (days) | 18.13 | 15.76-20.49 | 13.92 | 12.65-15.19 | -4.21 (-6.89--1.53) | 0.002 |

**Table3a: Outcomes for all patients**

|  | Warfarin group (n=538) | | NOAC group (n=175) | | Difference  （95%CI） | P |
| --- | --- | --- | --- | --- | --- | --- |
|  | Values | 95%CI | Values | 95%CI |  |  |
| After PSM (1:1) |  | | | | | |
| 30-day survival rate (%) | 84.03 |  | 73.57 |  |  | 0.04 |
| LOS ICU (days) | 9.43 | 7.78-11.08 | 6.46 | 5.41-7.50 | -2.97 (-4.93--1.01) | 0.003 |
| LOS Hospital (days) | 20.66 | 17.26-24.06 | 14.82 | 13.09-16.56 | -5.84 (-9.67--2.01) | 0.003 |

**Table3b: Outcomes for vasopressor subgroup**

LOS ICU: Length of ICU stay; LOS Hospital: Length of hospital stay; PSM: propensity score matching.
