# Supplementary figures and images for: Oral anticoagulants increased 30-day survival in sepsis patients complicated with atrial fibrillation: a retrospective analysis from MIMIC-IV database
Source: Front Cardiovasc Med. 2024 Jan 18;11:1322045. doi: 10.3389/fcvm.2024.1322045 (PMC10830619; doi:10.3389/fcvm.2024.1322045)

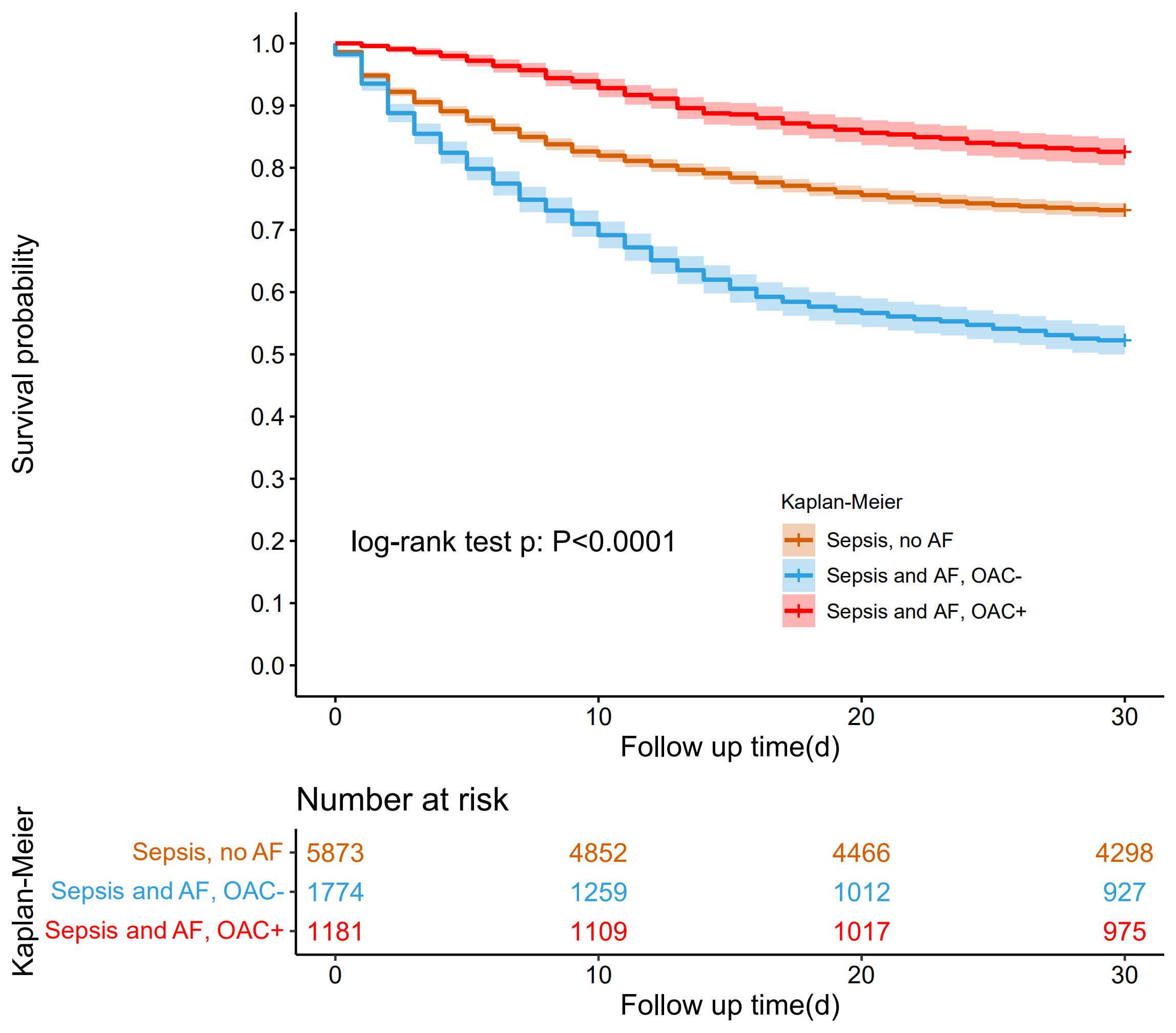

Supplement: Supplementary file 4 [file Image1.tif]

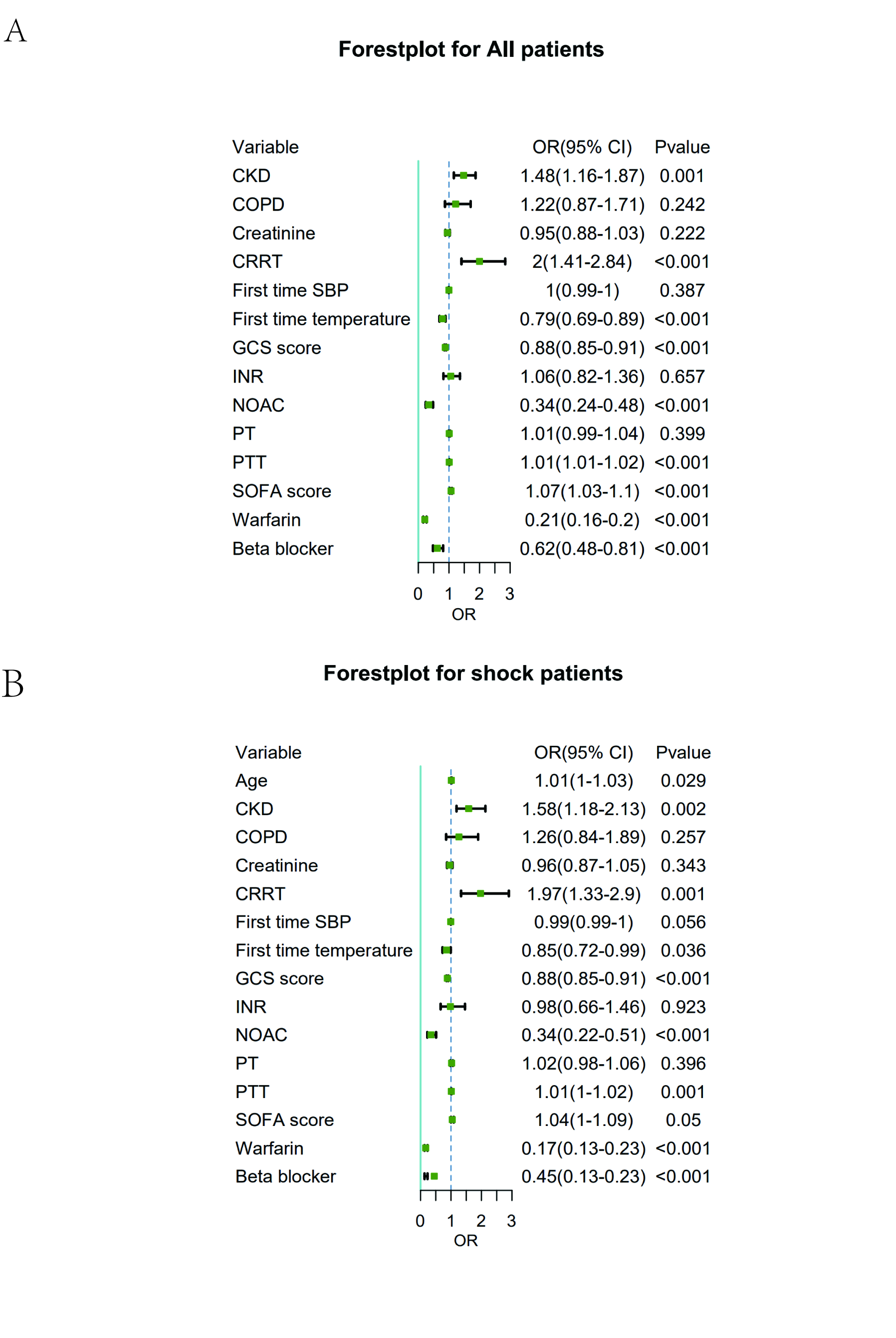

Supplement: Supplementary file 5 [file Image2.tif]
